# Ignored and undervalued in public health: a systematic review of health state utility values associated with syphilis infection

**DOI:** 10.1186/s12955-024-02234-1

**Published:** 2024-02-13

**Authors:** Patrick Miao, Fern Terris-Prestholt, Christopher K. Fairley, Joseph D. Tucker, Virginia Wiseman, Philippe Mayaud, Ying Zhang, Jane Rowley, Sami Gottlieb, Eline L. Korenromp, Caroline G. Watts, Jason J. Ong

**Affiliations:** 1 Central Clinical School, Monash University, Melbourne, Australia; 2 Department of Global Health and Development, London School of Hygiene and Tropical Medicine, London, United Kingdom; 3 UNAIDS, Geneva, Switzerland; 4 Melbourne Sexual Health Centre, Melbourne, Australia; 5 Faculty of Infectious and Tropical Diseases, London School of Hygiene and Tropical Medicine, London, United Kingdom; 6 University of North Carolina at Chapel Hill, Chapel Hill, USA; 7 Kirby Institute, University of New South Wales, Sydney, Australia; 8 Global HIV, Hepatitis and Sexual Transmitted Infections Programme, World Health Organization, Geneva, Switzerland; 9 Department of Sexual and Reproductive Health and Research, World Health Organization, Geneva, Switzerland; 10 The Daffodil Centre, The University of Sydney, a joint venture with Cancer Council NSW, Sydney, Australia; 11 Melbourne Sexual Health Centre, 580 Swanston Street, Carlton, Victoria 3053 Australia

**Keywords:** Syphilis, Health economics, Quality of life, Health state utility value, Utility weight, Disability weight, Disability-adjusted life year, Quality-adjusted life year

## Abstract

**Background:**

Syphilis is a sexually transmitted infection causing significant global morbidity and mortality. To inform policymaking and economic evaluation studies for syphilis, we summarised utility and disability weights for health states associated with syphilis.

**Methods:**

We conducted a systematic review, searching six databases for economic evaluations and primary valuation studies related to syphilis from January 2000 to February 2022. We extracted health state utility values or disability weights, including identification of how these were derived. The study was registered in the international prospective register of systematic reviews (PROSPERO, CRD42021230035).

**Findings:**

Of 3401 studies screened, 22 economic evaluations, two primary studies providing condition-specific measures, and 13 burden of disease studies were included. Fifteen economic evaluations reported outcomes as disability-adjusted life years (DALYs) and seven reported quality-adjusted life years (QALYs). Fourteen of 15 economic evaluations that used DALYS based their values on the original Global Burden of Disease (GBD) study from 1990 (published in 1996). For the seven QALY-related economic evaluations, the methodology varied between studies, with some studies using assumptions and others creating utility weights or converting them from disability weights.

**Interpretation:**

We found a limited evidence base for the valuation of health states for syphilis, a lack of transparency for the development of existing health state utility values, and inconsistencies in the application of these values to estimate DALYs and QALYs. Further research is required to expand the evidence base so that policymakers can access accurate and well-informed economic evaluations to allocate resources to address syphilis and implement syphilis programs that are cost-effective.

**Supplementary Information:**

The online version contains supplementary material available at 10.1186/s12955-024-02234-1.

## Introduction

Syphilis is caused by the sexually transmitted spirochete *Treponema pallidum* subspecies *pallidum*. Without timely treatment, disease in adults can progress from the early stage (primary and secondary syphilis) to the late stage (tertiary syphilis) causing severe cardiovascular and neurological disease and death [1]. Infection in pregnancy can result in stillbirth, neonatal death, prematurity, low birth weight, and congenital syphilis in neonates [2]. Global prevalence estimates of syphilis have remained steady thanks to expanded antenatal care coverage, however congenital syphilis is still a significant contributor to burden of disease in children [3, 4]. In some high-income countries like Australia and the United States, where eradication was once a public health prospect, syphilis cases are now resurging [5, 6]. Syphilis programs are substantially underfunded compared to nearly every other infectious disease, increasing the importance of economic evaluation studies to guide investment decision-making [7].

A common measurement of the impact of the disease is quality of life, often framed within the context of quality-adjusted life years (QALYs) or disability-adjusted life years (DALYs) [8]. Both measures combine the quality of life and duration lived in that state: the QALY is a measure of the amount of time lived in any given health state (measured by a utility weight) and the DALY is a combined measure of years lived with a disability (measured by a disability weight) or illness and the years of life lost – the values range from 0 to 1: for utility weights, perfect health is given a weighting of 1 and death is given a weighting of 0, and vice versa for disability weights. The Global Burden of Disease (GBD) projects remain dominant in developing health state utility values. The disability weight was introduced by the GBD team in the 1990s [9]. The disability weight methodology and the empirical data supporting it were substantially revised and updated for the 2010 iteration of GBD, and has received iterative updates to its evidence base in subsequent years [10].

Policymaking for syphilis screening and treatment programs, and investment in new tools for syphilis prevention and management, should be informed by up-to-date, accurate and well-designed economic evaluation studies. If health states are given weights that misrepresent real-life preferences, results can significantly underestimate or overestimate the cost-effectiveness of interventions [11]. The development of utility and disability weights for specific populations can be time-consuming and expensive [12]. In lieu of generating high-quality primary studies for a population, researchers commonly extrapolate using previously published utility and disability weights.

Systematic reviews on syphilis have focused on diagnostics and treatment [13, 14], but not on economic components. We aimed to summarise studies reporting utility and disability weights for health states associated with syphilis. Furthermore, we sought to locate the primary sources of these values and report how they were developed.

## Methods

We conducted a systematic review following the guidance from the Cochrane Handbook and Preferred Reporting Items for Systematic Reviews and Meta-Analyses (PRISMA) guidelines for reporting [15, 16]. The study was registered in the International Prospective Register of Systematic Reviews (PROSPERO, CRD42021230035).

### Inclusion criteria

The criteria for inclusion for economic evaluations were: the participants were men, women, or children with syphilis; the intervention was any program or procedure to prevent, control, or treat syphilis infection; the main outcomes were either cost-per-DALY or cost-per-QALY or the valuation of the health states associated with syphilis infection. For primary studies, we included them if a valuation for health state utility values was performed or if we found them as the primary source for a study. We did not explicitly search grey literature, though we did include government burden of disease studies referenced by included economic evaluations. The search was restricted to publications from January 1 2000 to February 4 2022. We excluded qualitative studies, studies without primary data, duplicates and studies not in English.

### Search strategy

Six databases (MEDLINE, EMBASE, NHS Economic Evaluation Database, Health Technology Assessment, Database of Reviews of Effects, Web of Science Core Collection) were searched on January 7, 2021, with an updated search on February 4, 2022 (see Supplementary Tables S1-7). The search was adapted from a systematic review valuing the health states associated with chlamydia [17]. Reference lists of studies were manually searched to find additional studies and to find the primary source of the utility or disability weights.

### Study selection

Two reviewers (PM, CW) independently screened the abstracts which met the inclusion criteria using Covidence systematic review software (Veritas Health Innovation, Melbourne, Australia). Any discrepancies were reviewed by a third reviewer (JO). Quality assessment or quality of reporting was conducted using the Consolidated Health Economic Evaluation Reporting Standards (CHEERS) checklist and the Consensus Health Economic Criteria (CHEC) checklist for economic evaluations, and an appraisal checklist by Picot for health-related quality-of-life primary studies (see Supplementary Tables S8-10) [18–20].

### Data extraction and analysis

Two reviewers (PM, CW) independently extracted data into an Excel spreadsheet. The data extracted from economic evaluations were author, year, country, study aims, study participants (gender, age, risk characteristics), study outcomes (DALY, QALY), health states related to syphilis, and utility or disability weights, and durations of disease. The data extracted from primary studies were the study population, methods used to calculate utilities, and health states valued with their results. The data extracted from the burden of disease studies were methods, the health states and their disability weights, and durations of disease. Descriptive statistics were used to summarise the characteristics of the included studies. Where information was lacking, we contacted one corresponding author who provided additional data.

## Results

**Fig. 1:**
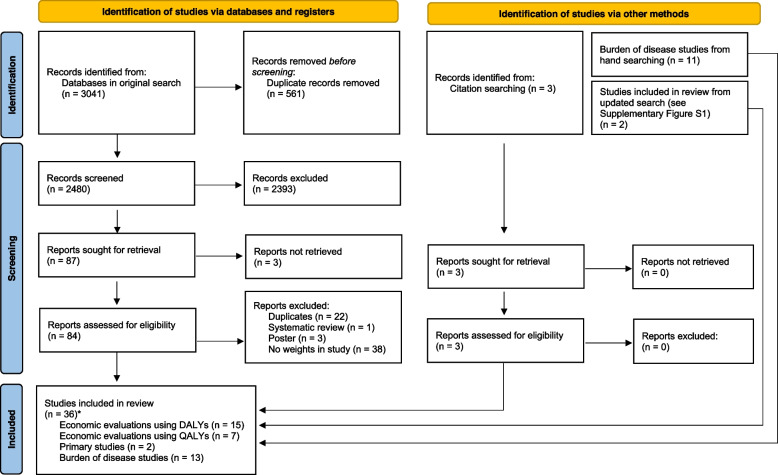
PRISMA flowchart of the search strategy. *one economic evaluation using QALYs was also a primary study and thus should only be counted once toward the total

Across the initial and updated searches, we returned 3401 studies (3041 from the initial search and 360 from the updated search). Following title and abstract screening, a total of 93 studies were selected for full-text review. Of these, 36 studies were included in our review. In total, 22 economic evaluations were included [21–40]; fifteen had the outcome framed in DALYs [21–34, 41], and seven had the outcome framed in QALYs [35–40, 42]. Two primary studies were identified after hand-searching reference lists [40, 43]. Note that one primary study was also an economic evaluation [40]. Thirteen burden of disease studies were included as part of the review: seven studies as part of the GBD series published by the Institute of Health Metrics and Evaluation [4, 44–49], four country-related landmark burden of disease studies [50–53], and two other separate burden of disease studies [54, 55]. Fig. 1 displays the PRISMA flowchart.

Supplementary Table S11 summarises the key characteristics of the 36 studies reviewed. For the 22 economic evaluations, two studies were from low-income countries [21, 27], three from lower-middle-income countries [32, 33, 41], three from upper-middle-income countries [22, 29, 31], eight from high-income countries [23, 35–40, 42], and six from a mixture of countries [24–26, 28, 30, 34]. Fifteen studies (68%) reported the cost-effectiveness of antenatal screening for syphilis [21, 22, 24–34, 36, 37]. In addition to studies reporting weights for mothers and their newborns, other common populations included blood donors and patients receiving blood transfusions [23, 41, 42], and people living with HIV [35, 39].

### Economic evaluations

Table 1 summarises the characteristics of the 22 economic evaluations. Of these, the primary outcome was the cost-per-DALY-averted in 15 studies and the cost-per-QALY-gained in seven studies. Health states reported in the text below and Tables 1, 2, 3, 4 and 5 reflect the choice of wording within the respective study (e.g. “mild early syphilis” or “stage one”), however with ambiguous terms we have standardised them to reflect the more widely accepted medical terminology.

**Table 1:** Key characteristics of health economic evaluations related to syphilis infection

**Lead author**	**Evaluation aims**	**Country**	**Population targeted by the intervention**	**Health states included**	**Source for disability weights or utility weights**
**Primary outcome: DALYs**					
Bristow (2016) [21]	Assess the health and economic outcomes of a dual testing strategy in a simulated cohort of antenatal care patients in Malawi	Malawi	Women attending antenatal clinic	Congenital syphilis, low birth weight, neonatal death, stillbirth	[76]
Hong (2010) [22]	Assess the effectiveness of a program preventing mother-to-child transmission of syphilis	China	Women attending antenatal clinic	Congenital syphilis	[44, 77]
Jayawardena (2019) [23]	Analyse alternative options for donor syphilis testing to determine the optimal strategy	Australia	Blood donors	Adult early syphilis, adult tertiary syphilis, congenital syphilis	[45, 78] Durations from [79]
Kahn (2014) [24]	Assess the cost-effectiveness of scaling-up syphilis screening and treatment in existing antenatal care programs in various contexts	Modelled scenarios in generic countries	Women attending antenatal clinic	Congenital syphilis, low birth weight, stillbirth, neonatal death	[76]
Kuznik (2013) [25]	Evaluate the cost-effectiveness and budget impact of antenatal syphilis screening for 43 countries in sub-Saharan Africa and estimate the impact of universal screening on outcomes	43 sub-Saharan African countries	Women attending antenatal clinic	Congenital syphilis, stillbirth, neonatal death	[45]
Kuznik (2015) [26]	Evaluated the cost-effectiveness of increasing the coverage for antenatal syphilis screening in 11 Asian and 20 Latin American countries using a POC test compared to no testing and no treatment	11 Asian and 20 Latin American countries	Women attending antenatal clinic	Congenital syphilis, stillbirth, neonatal death	[80]
Larson (2014) [27]	Estimate the costs and cost-effectiveness of using rapid syphilis POC tests compared to usual care	Zambia	Women attending antenatal clinic	Congenital syphilis, low birth weight, stillbirth, neonatal death	[24]
Owusu-Edusei (2011) [28]	Compare the health and economic outcomes of a dual nontreponemal-treponemal point-of-care test with existing syphilis tests/testing algorithms in a high prevalence setting	Sub-Saharan Africa	Women attending antenatal clinic	Adult early syphilis, adult tertiary syphilis, congenital syphilis, low birth weight, miscarriage, stillbirth, neonatal death	[64]
Owusu-Edusei (2014) [29]	Compare the economic and health outcomes of four new antenatal HIV and syphilis screening strategies	China	Women attending antenatal clinic	Adult early syphilis, adult tertiary syphilis, HIV-positive women with syphilis coinfection, congenital syphilis, low birth weight, stillbirth, induced abortion for infected mothers, neonatal death, HIV in newborn	[45]
Rodriguez (2021) [30]	Assess the cost-effectiveness of dual testing during antenatal care in four countries with varying HIV and syphilis prevalence	South Africa, Kenya, Colombia, Ukraine	Women attending antenatal clinic	Congenital syphilis, low birth weight, stillbirth, neonatal death	[10]
Romero (2020) [31]	Assess the cost-effectiveness of a rapid point-of-care test and treatment if required compared with a laboratory-based standard test with treatment at the next follow-up visit	Brazil	Women attending antenatal clinic	Congenital syphilis, low birth weight, stillbirth, neonatal death	[54]
Russell (2021) [41]	Estimate the cost-effectiveness of testing all blood donations for HIV, hepatitis B and C, and syphilis to avoid adverse health outcomes in recipients	Ghana	Patients receiving blood transfusions	Acute syphilis infection	[42]
Schackman (2007) [32]	Estimate the cost-effectiveness, projected health outcomes, and the annual cost of screening pregnant women using a rapid syphilis test	Haiti	Women attending antenatal clinic	Congenital syphilis, stillbirth, neonatal death	[64]
Terris-Prestholt (2003) [33]	Estimate the cost-effectiveness of on-site antenatal syphilis screening and treatment	Tanzania	Women attending antenatal clinic	Low birth weight, stillbirth	[44]
Terris-Prestholt (2015) [34]	Assess the cost-effectiveness of clinic-based rapid plasma reagin and single rapid syphilis test in 20 antenatal clinics across Peru, Tanzania, and Zambia	Peru, Tanzania, Zambia	Women attending antenatal clinic	Congenital syphilis, low birth weight, stillbirth, neonatal death	Partially from [25]
**Primary outcome: QALYs**					
Castillo (2021) [40]	Develop a modelling framework for cost-effectiveness evaluation of different approaches using rapid tests for the detection of syphilis in inmates’ populations	Chile	Prisoners	Adult syphilis infection (susceptible, stage 1, stage 2, latent, tertiary, immune)	[81, 82]
Custer (2010) [42]	Assessed the cost-effectiveness of pathogen reduction technology in mitigating the risk of transfusion-associated infectious and some non-infectious threats	Canada	Patients receiving blood transfusions	Primary syphilis, tertiary syphilis	Assumption for primary syphilis; For tertiary syphilis [57]
Eaton (2018) [35]	Compare the cost-effectiveness of the reverse and traditional syphilis screening algorithms in persons living with HIV	United States	People living with HIV	HIV and syphilis infection	[39]
Hersh (2018) [36]	Estimate the cost-effectiveness of screening all women during the first and third trimesters compared with screening just once during pregnancy	United States	Women attending antenatal clinic	Congenital syphilis, intrauterine fetal demise, neonatal death, congenital syphilis (maternal perspective), intrauterine fetal demise (maternal perspective), neonatal death (maternal perspective)	For fetal perspectives [58] For maternal perspectives [83, 84]
Huntington (2020) [37]	Assess the cost-effectiveness of universal repeat screening for syphilis in late pregnancy, compared with the current strategy of single screening in early pregnancy with repeat screening offered only to high-risk women	United Kingdom	Women attending antenatal clinic	Congenital syphilis, intrauterine fetal demise, neonatal death	[58]
Suijkerbuijk (2018)^a^ [38]	Evaluate intended savings and missed syphilis and/or HIV infections and explore the efficiency of possible test policies	Netherlands	People attending a sexual health clinic	Syphilis	[53]
Tuite (2014)^a^ [39]	Evaluate the cost-effectiveness of strategies that increased the frequency and population coverage of syphilis screening in HIV-infected men who have sex with men receiving HIV care relative to the current standard of care	Canada	Men who have sex with men living with HIV	Primary syphilis, secondary syphilis, neurosyphilis and tertiary syphilis	[45, 85]

*POC* Point of care, *HIV* Human immunodeficiency virus

^a^Studies used disability weights for valuation of health states but calculated their outcomes in terms of QALYs

**Table 2:** Disability weights for health economic evaluations where the primary outcome was measured in DALYs

**Study**	**Neonatal**			**Adult**		
	**Health state**	**Disability weight**	**Duration**	**Health state**	**Disability weight**	**Duration**
Bristow (2016) [21]	Congenital syphilis	0·315	3 years	··	··	··
	Low birth weight or prematurity	0·106	1 year	··	··	··
	Miscarriage	0	··	··	··	··
	Stillbirth	0	··	··	··	··
	Neonatal death	1	··	··	··	··
Hong (2010) [22]	Congenital syphilis	0·315	3 years	··	··	··
	Fetal and neonatal death (including neonatal death due to low birth weight, neonatal death due to congenital syphilis, perinatal death, spontaneous abortion, medical/induced abortion)	1	··	··	··	··
Jayawardena (2019) [23]	··	··	··	Mild early syphilis (primary or secondary)	0·006 (0·002-0·012)	0·07 years
	··	··	··	Tertiary syphilis	0·203 (0·134-0·29)	10 years
	Congenital syphilis	0·315	3 years	··	··	··
Kahn (2014) [24]	Congenital syphilis	0·316	Lifetime	··	··	··
	Low birth weight	0·106	Lifetime	··	··	··
	Stillbirth	Calculated as a set number of DALYs per stillbirth – 4·95 DALYs	··	··	··	··
	Neonatal death	Calculated as a set number of DALYs per neonatal death – 9·4 DALYs				
Kuznik (2013) [25]	Congenital syphilis	0·315 (0·159-0·471)	Lifetime	··	··	··
	Stillbirth	1	··	··	··	··
	Neonatal death	1	··	··	··	··
Kuznik (2015) [26]	Congenital syphilis	0·315 (0·159-0·471)	Not specified	··	··	··
	Stillbirth	1	··	··	··	··
	Neonatal death	1	··	··	··	··
Larson (2014) [27]	Congenital syphilis	0·316	Lifetime	··	··	··
	Low birth weight	0·106	Lifetime	··	··	··
	Stillbirth	1	··	··	··	··
	Neonatal death	1	··	··	··	··
Owusu-Edusei (2011) [28]	··	··	··	Early syphilis (primary or secondary)	0·032 (0·015-0·048)	Not specified
	··	··	··	Tertiary syphilis	0·283 (0·250-0·300)	Not specified
	Congenital syphilis	0·315 (0·250-0·350)	3 years	··	··	··
	Low birth weight	0·106 (0·090-0·130)	1 year	··	··	··
	Miscarriage	1	··	··	··	··
	Stillbirth	1	··	··	··	··
	Neonatal death	1	··	··	··	··
Owusu-Edusei (2014) [29]	··	··	··	Early (primary or secondary) syphilis	0·015 (0·0075-0·0225)	Not specified
	··	··	··	Tertiary syphilis	0·283 (0·01415-0·4245)	Not specified
	··	··	··	HIV-positive pregnant women with syphilis coinfection	0·38	Not specified
	Congenital syphilis	0·315 (0·1575-0·47)	3 years	··	··	··
	Low birth weight	0·106 (0·053-0·159)	1 year	··	··	··
	Induced abortion	0	··	··	··	··
	Stillbirth	1	··	··	··	··
	Neonatal death	1	··	··	··	··
Rodriguez (2021) [30]	Congenital syphilis	0·315	3 years	··	··	··
	Low birth weight	0·106	1 year	··	··	··
	Stillbirth	1	First 20 years of life	··	··	··
	Neonatal death	1	First 20 years of life	··	··	··
Romero (2020) [31]^a^	Congenital syphilis	0·315	Lifetime	··	··	··
	Low birth weight	0·106	Lifetime	··	··	··
	Stillbirth	1	··	··	··	··
	Neonatal death	1	··	··	··	··
Russell (2021) [41]	··	··	··	Syphilis	0·12 (0·09-0·15)	90 days
Schackman (2007) [32]	Congenital syphilis	0·315 (0·1575-0·4725)	Lifetime	··	··	··
	Stillbirth	1	··	··	··	··
	Neonatal death	1	··	··	··	··
Terris-Prestholt (2003) [33]	Low birth weight	0·291	Lifetime	··	··	··
	Stillbirth	1	··	··	··	··
Terris-Prestholt (2015) [34]	Congenital syphilis	0·315	Lifetime	··	··	··
	Low birth weight	0·291	Lifetime	··	··	··
	Stillbirth	1	··	··	··	··
	Neonatal death	1	··	··	··	··

Ranges or confidence intervals used for sensitivity analyses are placed in brackets

*DALY* Disability-adjusted life year, *HIV* Human immunodeficiency virus

^a^Weights not explicitly stated in the study but are taken from a paper referenced in the bibliography [25]

**Table 3:** Syphilis-related health states and utility weights used in cost-effectiveness studies where the primary outcome was QALYs

**Study**	**Neonatal**			**Adult**		
	**Health state**	**Utility weight**	**Duration**	**Health state**	**Utility weight**	**Duration**
Castillo (2021) [40]	··	··	··	Susceptible	1	-
	··	··	··	Stage 1 (primary)	0·737	4·93 years
	··	··	··	Stage 2 (secondary)	0·737	3·38 years
	··	··	··	Latent	0·737	0·33 years
	··	··	··	Tertiary	0·737	-
	··	··	··	Immune	1	0·2 years
Custer (2010) [42]	··	··	··	Primary syphilis	0·88 (0·85-0·91)	1 year
				Tertiary syphilis	0·65 (0·6-0·70)	Not stated
Eaton (2018) [35]	··	··	··	Syphilis and HIV infection	0·82 (0·69-0·93)	1 year if positive screening test, 2 years if false negative screening test
Hersh (2018) [36]	Congenital syphilis	0·74 (0·6-0·8)	Lifetime	··	··	··
	Intrauterine fetal demise	0	··	··	··	··
	Neonatal death	0	··	··	··	··
	··	··	··	Congenital syphilis (maternal perspective)	0·88 (0·7-0·9)	Lifetime
	··	··	··	Stillbirth (maternal perspective)	0·92 (0·8-0·95)	Lifetime
	··	··	··	Neonatal death (maternal perspective)	0·76 (0·7-0·8)	Lifetime
Huntington (2020) [37]	Congenital syphilis	0.74	Lifetime	··	··	··
	Intrauterine fetal demise	0	··	··	··	··
	Neonatal death	0	··	··	··	··
Suijkerbuijk (2018)^a^ [38]	··	··	··	Primary syphilis	1-0·015 = 0·985^b^ (age 1-44 years) 1-0·014 = 0·986^b^ (age 45+ years)	0·04 years
	··	··	··	Secondary syphilis	1-0·048 = 0·952^b^ 1-0·044 = 0·956^b^	0·07 years
	··	··	··	Neurosyphilis	1-0·281 = 0·719^b^	10 years
Tuite (2014) [39]	··	··	··	Primary syphilis	1-0·0072 = 0·9928^b^ (0·0065-0·0079)	0·7 years
	··	··	··	Secondary syphilis	1-0·041 = 0·959^b^ (0·036-0·045)	3·6 years
	··	··	··	Neurosyphilis and tertiary syphilis	1-0·094 = 0·906^b^ (0·074-0·283)	7·7 years

^a^Results are taken from the study referenced in the bibliography (State of Infectious Diseases in the Netherlands 2013)

^b^Disability weights were converted into utility weights by using the formula: 1 minus disability weight = utility weight

Ranges or confidence intervals used for sensitivity analyses are placed in brackets

**Table 4:** Primary studies with utility weights used in economic evaluations evaluating the impact of syphilis infections

**Lead author**	**Study aims**	**Participant characteristics**	**Participant age**	**Number of participants**	**Techniques used**	**Health states valued and utility weights**
Bennett (2000) [43]	Describe parents' values for outcomes of acute occult bacteraemia using utility assessment	Parents presenting to paediatric ED in an urban children's hospital with a child between 3-36 months	27·8 (SD 6·6)	94	Visual analogue scale, followed by chained standard gamble	Meningitis with minor brain damage = 0·7393
Castillo (2021) [40]	Modelling cost-effectiveness of syphilis detection strategies in prisons in Chile	Chileans living in metro areas	46·83 (SD 0·4)	1695	Interviews from two populations of syphilis patients were combined and applied to a Chilean EQ-5D valuation	Susceptible = 1; Any of infected stage 1, infected stage 2, latent or tertiary = 0·737; Immune = 1

*SD* Standard deviation, *EQ-5D* EuroQol-5 Dimensions

**Table 5:** Disability weights for syphilis-related conditions in Global Burden of Disease Studies^a^ and Related Studies

**Study**	**Methods**	**Sequela**	**Mapped health state**	**Disability weight**	**Ranges or 95% confidence intervals**	**Stated source for disability weight**	**Disease duration**	**Source for duration**
**Global Burden of Disease**								
Global Burden of Disease 1990 [44]; Global Burden of Disease 1990 (2004 update) [45]	Time trade-off: small groups of health professionals performed weighting exercises to make a composite judgment on the severity distribution of the condition and the social preference for time spent in each severity level	Congenital syphilis	··	0·315	··	··	··	··
		Low birth weight	··	0·106	··	··	··	··
		Primary syphilis	··	0·014 (1-44 years) 0·015 (45+ years)	··	··	··	··
		Secondary syphilis	··	0·048 (1-59 years) 0·044 (60+ years)	··	··	··	··
		Tertiary syphilis	··	0·283	··	··	··	··
Global Burden of Disease 2019 [4]	Disability weights have been generated using data collected from more than 31 000 respondents through population-based surveys in multiple countries and an open internet survey	Mild early syphilis infection	Infectious disease, acute episode, mild	0·006	0·002-0·012	··	··	··
		Asymptomatic early syphilis infection	Asymptomatic	0	-	··	··	··
		Cardiovascular complications due to adult tertiary syphilis	Infectious disease, acute episode, moderate	0·051	0·032-0·074	··	··	··
		Severe disfigurement due to adult tertiary syphilis	Disfigurement, level 3	0·405	0·275-0·546	··	··	··
		Neurological problems due to adult tertiary syphilis	Motor plus cognitive impairments, moderate	0·203	0·134-0·29	··	··	··
		Asymptomatic adult tertiary syphilis	Asymptomatic	0	-	··	··	··
		Neurological problems and cardiovascular complications due to adult tertiary syphilis	Moderate motor plus cognitive impairments and moderate infectious disease, acute episode	0·243	0·168-0·333	··	··	··
		Severe disfigurement and cardiovascular complications due to adult tertiary syphilis	Level 3 disfigurement and moderate infectious disease, acute episode	0·435	0·306-0·571	··	··	··
		Severe disfigurement and neurological problems due to adult tertiary syphilis	Level 3 disfigurement and moderate motor plus cognitive impairments	0·523	0·378-0·669	··	··	··
		Severe disfigurement, neurological problems, and cardiovascular complications due to adult tertiary syphilis	Level 3 disfigurement, moderate motor plus cognitive impairments, and moderate infectious disease, acute episode	0·547	0·402-0·691	··	··	··
**Related Studies**								
The Burden of Disease and Injury in Australia 1999 [50]	Taken from GBD 1990	Primary syphilis	··	0·148	··	GBD 1990	··	··
		Secondary syphilis	··	0·048	··		··	··
		Tertiary syphilis (cardiovascular)	··	0·196	··		··	··
		Tertiary syphilis (gummas)	··	0·102	··		··	··
		Tertiary syphilis (neurologic)	··	0·283	··		··	··
		Congenital syphilis	··	0·315	··		··	··
Victorian Burden of Disease Study 2001 [51]	Taken from GBD 1990	Secondary syphilis	··	0·048	··	GBD 1990	··	··
		Tertiary syphilis (cardiovascular)	··	0·196	··		··	··
		Tertiary syphilis (gummas)	··	0·102	··		··	··
		Tertiary syphilis (neurologic)	··	0·283	··		··	··
		Congenital syphilis	··	0·315	··		··	··
		Congenital syphilis	··	0·315	··		··	··
Ontario Burden of Infectious Disease 2010 [52]	Uses a Canadian-developed combination of scores for 11 attributes (using a standard gamble) with predominantly lay panels to develop a severity weight by subtracting the preference weight from 1·0^a^	Primary syphilis	··	0·017	··	Primary source	14 days	The Burden of Disease and Injury in Australia 1999 [50]
		Secondary syphilis	··	0·039	··		28 days	
		Neurosyphilis	··	0·074	··		10 years	
		Congenital syphilis	··	0·139	··		3 years	
State of Infectious Diseases in the Netherlands 2013 [53]	Taken from GBD 1990	Primary syphilis	··	0·014 (1-44 years) 0·015 (45+ years)	··	GBD 1990	··	··
		Secondary syphilis	··	0·048 (1-59 years) 0·044 (60+ years)	··		··	··
		Neurosyphilis	··	0·281	··		··	··
		Congenital syphilis	··	0·315	··		··	··
Kuznik 2015 [54]	Estimate the public health burden resulting from adverse pregnancy outcomes due to syphilis infection among pregnant women not screened for syphilis in 43 countries in sub-Saharan Africa	Congenital syphilis	··	0·315	··	GBD 1990	Lifetime	··
		Low birth weight	··	0·106	··		Lifetime	··
		Stillbirth	··	1	··		··	··
		Neonatal death	··	1	··		··	··
Liu 2018 [55]	Estimate the disease burden resulting from syphilis-related adverse pregnancy outcomes in Hunan Province and assess the progress of a free syphilis screening and treatment program	Congenital syphilis	··	0·315	··	GBD 1990 (2004 update)	Lifetime	··
		Low birth weight	··	0·106	··		Lifetime	··
		Miscarriage	··	1	··		··	··
		Stillbirth	··	1	··		··	··
		Neonatal death	··	1	··		··	··

Global Burden of Disease studies for 2010, 2013, 2015, and 2017 have been omitted [46–49], because they are added to each iteration, culminating in the 2019 values (see Supplementary File S3)

Data found from Global Health Data Exchange at http://ghdx.healthdata.org/ run by the Institute for Health Metrics and Evaluation (IHME) [86]

*GBD* Global Burden of Disease, *CLAMES* Classification and Measurement System of Functional Health

^a^Uses a “severity weight”, which is conceptually similar to a disability weight – that is, the higher the weight, the “worse” the health state

#### DALYs

Table 2summarises the disability weights and associated durations used in the 15 economic evaluations in which the primary outcome was framed in DALYs [21–34, 41]. The commonly used health states for newborns were congenital syphilis (*n*=13) with disability weight ranges from 0·315 to 0·316, low birth weight (*n*=9) with ranges from 0·106 to 0·291, and miscarriage (*n*=3), stillbirth (*n*=13), and neonatal death (*n*=12) all with values of 0 or 1. Health states for adult syphilis included early syphilis (*n*=4), tertiary syphilis (*n*=4), and HIV and syphilis coinfection (*n*=1), with disability weight ranges from 0·006 to 0·38. Eleven economic evaluations sourced their disability weights directly from a Global Burden of Diseases study [21–26, 28, 29, 31–33]. Of these eleven studies, only one used weights from after the 2010 update [23]. Three studies referenced other economic evaluations in our review for their disability weights [27, 30, 34]. The only economic evaluation that did not use GBD weights assumed the health state utility value, giving a disability weight of 0·12 for syphilis [41].

Eleven studies used a disability weight of 0·315 for clinical congenital syphilis [21–23, 25, 26, 28–32, 34], while two used a weight of 0·316 [24, 27]. One of the studies which used 0·316 referenced the other study [27, 56]. Six studies applied the disability weight for three years [21–23, 28–30], five applied the disability weight over the life expectancy of the newborn [24, 25, 27, 32, 34], and two did not specify the applied duration of disability [26, 31].

Nine studies contained disability weights for low birth weight as a sequelae of congenital syphilis [21, 24, 27–31, 33, 34], seven of which used a disability weight of 0·106 [21, 24, 27–31]. Two studies by the same author used a disability weight of 0·291 [33, 34]. Four studies applied the disability weight for one year [21, 28–30], and five studies applied the disability weight over the life expectancy of the newborn [24, 27, 31, 33, 34].

Thirteen studies included stillbirth and/or neonatal death attributed to syphilis [21, 22, 24–34]. In ten studies, stillbirths and neonatal deaths were counted as a full discounted life expectancy lost due to disability (i.e. disability weight of 1) [22, 25–29, 31–34]. Of the other three studies, one study gave neonatal death a disability weight of 1, but gave stillbirth a disability weight of 0 [21]; one study converted stillbirths and neonatal deaths to a set quantity of DALYs, calculating 4·95 DALYs per stillbirth and 9·4 DALYs per neonatal death [24]; another study applied years of life lost from stillbirths and neonatal deaths only up to 20 years of age with discounting [30]. Three studies listed miscarriage as an adverse pregnancy outcome [21, 22, 28], and two of these studies gave equal weight to stillbirth and neonatal death (i.e. disability weight = 1) [22, 28], with one study valuing miscarriage as 0 (i.e. no disability) [21]. Two studies included induced abortion as an adverse pregnancy outcome, with one study giving it a disability weight of 1 [22], and another study giving it a disability weight of 0 [29].

Four economic evaluations contained disability weights for adult syphilis [23, 28, 29, 41], using results from editions of the GBD from 1990 to 2015. The most recent of the three studies, published in 2019, used disability weights of 0·006 for mild early syphilis and 0·203 for tertiary syphilis which were taken from the 2015 GBD study [23]. One study used a disability weight of 0·12 and referenced a paper in which the utility weight for adult syphilis of 0·88 was an assumption; the duration of 90 days for adult syphilis infection was also assumed [41, 42].

#### QALYs

Table 3 summarises the utility weights used in the seven economic evaluations in which the primary outcome was framed in QALYs [35–40, 42]. Utility weights among these papers ranged from 0.65 to 0.9928. Four studies applied utility weights only for adults with syphilis [35, 38–40, 42], one study for newborns only [37], and one study for both adults and newborns [36]. One study applied the same utility weight of 0·737 regardless of disease stage (stage 1, stage 2, latent, tertiary) but varied the mean duration by stage [40]. One study used a utility weight of 0·82 for syphilis and HIV coinfection [35]; however, the referenced source did not contain the weight [39]. One study assessing transfusion recipients gave a utility weight assumption for primary syphilis of 0·88, and used a utility weight of 0·65 for tertiary syphilis taken from a catalogue which could not be verified [42, 57].

One study included a maternal perspective in the analysis, applying a reduced quality of life for women experiencing a stillbirth, neonatal death, or giving birth to a child with congenital syphilis, with utility weights of 0·92, 0·76, and 0·88 respectively, and applied these weights to a lifetime duration to generate QALYs for the mother [36].

Two studies used an adapted utility weight of 0·74 for congenital syphilis [36, 37]; this was based on studies valuing outcomes – specifically meningitis – from occult bacteraemia in children (i.e. bacteria in the bloodstream without an obvious source of infection) given the lack of a known utility weight for congenital syphilis [43, 58].

The duration for which utility weights were applied varied between all seven studies. One study applied a duration of 4·93 years for stage 1, 3·38 years for stage 2, 0·33 years for a latent period, and 0·2 years for an immune period [40]. One study which converted utility weights from disability weights used durations of 0·04 years for primary syphilis, 0·07 years for secondary syphilis, and 10 years for neurosyphilis [38]. Another study which converted utility weights from disability weights used durations of 0·7 years for primary syphilis, 3·6 years for secondary syphilis, and 7·7 years for neurosyphilis and tertiary syphilis [39]. Two studies applied all utility weights over a lifetime time horizon [36, 37]. One study assumed that if a syphilis diagnosis was missed it would be detected within the subsequent year (thus resulting in an implied duration of up to two years), and another study looking at transfusion-related transmission assumed that primary syphilis would be treated in the year following transfusion (giving an implied duration of one year) [35, 42].

Two studies framed their outcome in QALYs but used disability weights instead of utility weights to calculate QALYs [38, 39]. One study used unique disability weights to other economic evaluations included in this review: 0·0072 for primary syphilis, 0·041 for secondary syphilis, and 0·094 for tertiary syphilis [39]. One study looked at the cost-savings of removing certain sexually transmitted infections from a routine screening panel and reported cost savings per QALY lost – the QALY loss per missed adult syphilis infection was calculated to be 0·005 [38].

Half of the economic evaluations provided ranges or confidence intervals for disability or utility weights which were used in sensitivity analyses. The ranges for disability weights for the seven out of fifteen DALY studies [23, 25, 26, 28, 29, 32, 41], as well as the ranges for the utility weights for the four out of seven QALY studies are provided in Tables 2 and 3 [35, 36, 39, 42].

### Primary sources and methods

Table 4 summarises the two primary sources for weights [40, 43]. The first primary source was a valuation study in which Canadian parents were asked to value health states associated with bacterial meningitis using a combination of standard gamble and visual-analogue scale. A condition-specific utility weight of 0·74 was created for “meningitis with minor brain damage”, and was subsequently used in a separate study analysing newborn screening strategies – in this study, the weight of 0·74 was applied to a health state named “mild developmental delay”. This health state and its weight was used by two separate economic evaluations to represent congenital syphilis [36, 37].

The second primary study developed its own utility weight by adapting the EuroQol-5 Dimensions (EQ-5D) questionnaire and conducting interviews with two groups: 29 syphilis-affected inmates and 67 patients from a sexually transmitted infections outpatient clinic. The mean utility weights of the two populations for primary, secondary, tertiary, and latent syphilis were 0.737. It then subsequently used the values in its own economic evaluation [40]. Further details of the methodology for developing utility weights for the primary sources can be found in Supplementary File S1.

### Global burden of disease and related studies

The Global Burden of Disease project comprises seven major publications of disability weights spanning GBD 1990 and GBD 2019 [4, 44–49]. A minor GBD update (GBD 2016) was published as an update to GBD 2015 and does not provide revisions to syphilis disability weight data and so was not included in this review [59].

In 1990 and its corresponding 2004 update, the GBD team developed disability weights in small focus groups [44]. The initial set of disability weights were: 0·315 for congenital syphilis, 0·015 for primary syphilis, 0·048 for secondary syphilis, 0·283 for tertiary syphilis, and 0·106 for low birth weight. In GBD 2010 [4, 46–49], the methodology for deriving disability weights was substantially revised, using population-based surveys, wherein respondents are presented with pairs of narrative generic health state descriptions and asked to say which of two individuals they consider healthier. GBD researchers modelling the burden of specific diseases and injuries then choose the narrative generic health states that best match specific disease outcomes based on clinical expert opinion. The population-based disability weight for that generic health state is applied to the specific disease outcome. This approach seeks to value outcomes such as disfigurement or cognitive impairment equally regardless of the disease or injury responsible [10]. The suite of generic health states for which disability weights have been estimated has been expanded with subsequent surveys [60, 61], and with each new GBD publication health state assignments may be changed or combined. Combinations include, for example, “severe disfigurement and cardiovascular complications due to adult tertiary syphilis”, as a combination of the health states of “Level 3 disfigurement” and “moderate infectious disease, acute episode”, which is calculated to have a disability weight of 0·435 [10, 60]. In GBD 2019, there are ten unique health states relating to syphilis, all of which are for adult syphilis [4].

It is not uncommon for studies calculating the burden of syphilis to adopt earlier published health state utility values (and in the case of congenital syphilis and low birth weight, if authors wish to use GBD values, then they must be from prior to GBD 2010). The Victorian Burden of Disease Study 2001 [51], the Burden of Disease and Injury in Australia study 1999 [50], the State of Infectious Diseases in the Netherlands 2013 [53], Kuznik’s burden of disease study in sub-Saharan Africa in 2015 [54], and Liu’s burden of disease study in China in 2018 [55], all use the same weights as those in the initial Global Burden of Disease study in 1990. No studies provided estimations or recommendations for the durations of the different health states.

Only one related study, the Ontario Burden of Infectious Disease 2010 [52], provides new disability weights using a new quality of life instrument. For syphilis, severity weights were developed for four health states: primary syphilis (0·017), secondary syphilis (0·039), neurosyphilis (0·074), and congenital syphilis (0·139). These studies are summarised in Table 5.

## Discussion

This systematic review summarised the health state utility and disability weights for syphilis. To our knowledge, this is the first systematic review of the health sequelae of syphilis infection. Similar systematic reviews have been performed for chlamydia [17], genital warts [62], and genital herpes [63]. Our findings show an overreliance on the same few weights, a lack of transparency when reporting how weights are derived, and inconsistency when applying these weights with regard to the values of the weights, the clinical stages, and the respective duration over which they are applied.

Disability weights, used as part of the calculation for DALYs, were applied in 15 of the 22 economic evaluations in our review. Fourteen studies based their weights on the 1990 Global Burden of Disease study [21–34, 64]. The methodology for developing the initial disability weights is described in Supplementary File S2. This methodology was criticised for its lack of transparency and on ethical and distributional grounds [65–68]. The methodology was substantially revised in response to these criticisms in GBD 2010, but economic evaluations of syphilis have generally not taken up the modern GBD disability weights, which are based on population-based surveys and generic health state descriptions, rather than focus groups with disease-specific expertise [10]. Aside from the weights, we found inconsistency in the duration of both disability and utility weights. For example, of 11 congenital syphilis-related studies, six applied the weighting for only three years [21–23, 28–30], and the other five applied the weight over the life expectancy of the newborn [24, 25, 27, 32, 34]. For a single case, the difference between using a disability weight for three years and a lifetime is significant (without discounting or age weighting, as per GBD 2010 onward) [67]. As an example, 0·315 multiplied by three years gives 0·945 years lost to disability for a single case, whereas 0·315 multiplied by a life expectancy of 65 yields 20·475 years lost to disability. There was a similar finding for utility weights. Of six QALY-related economic evaluations relating to adult syphilis, there were six unique sets of durations for syphilis health states ranging from weeks to total life expectancy [35, 36, 38–40, 42].

Our study highlights the limited evidence base for utility and disability weights and the durations they are applied for syphilis. Four economic evaluations explicitly stated a lack of validated evidence for syphilis utility weights may have led to uncertainty in their results [22, 36–38]. For such an uncertain variable, only half of the economic evaluations used ranges for disability or utility weights in their sensitivity analyses [23, 25, 26, 28, 29, 32, 35, 36, 39, 41, 42]. Outside of the GBD studies, we found only two primary sources for syphilis utility weights which developed their own utility weights for burden of disease calculation: one for congenital syphilis and one for adult syphilis [40, 43]. The congenital syphilis weights come from a study of meningitis in children [43]; this was used in two syphilis economic evaluations with no biomedical justification for equating congenital syphilis with childhood meningitis [36, 37]. A primary study for adult syphilis reflects a similar lack of evidence base for utility weights [40]. The study combined two Chilean prison populations (combined sample of 96 adults) to produce a single weight (0·737) that they applied to all forms of adult syphilis, from the primary stage to the life-threatening tertiary stage.

The disability weights and duration estimates constructed by the Global Burden of Disease project for the 1990 estimates have been updated. Salomon undertook work to update the weights for the 2010 GBD using a new methodology which addressed some of the concerns levelled at the original GBD 1990 methodology (see Supplementary File S3) [10, 60]. The interventions in many of the studies related to antenatal care, thus congenital syphilis and its sequelae were the most common health states to be valued in our review with a total of sixteen out of 22 economic evaluations doing so. However, there is little guidance in the updated disability weights as to which health states should be used when calculating the burden of childhood health states, such as for “congenital syphilis” and “low birth weight”, which no longer exist in GBD studies from 2010 onward [4]. For example, the 2019 GBD presents hundreds of congenital health sequelae that use niche wording such as “moderate hearing loss with ringing due to other congenital anomalies”. Given the myriad of clinical presentations associated with congenital syphilis [69, 70], guidance on the selection or pooling of relevant health states is needed to ensure studies take a consistent approach to the selection of weights for their models. We hope that future GBD studies reintroduce congenital syphilis and its sequelae to aid researchers in representing quality-of-life loss in these populations. For this systematic review, despite there being twelve economic evaluations using DALYs published after 2010, only one study used weights taken from after the GBD 2010 update [23]. Of three studies published after 2018 using GBD weights, two economic evaluations and one burden of disease study published have not used the GBD’s updated weights and have instead used the GBD 1990 weights [30, 31, 55]. Within the economic evaluations, there was minimal discussion or justification of the use of older weights, though the lack of congenital syphilis weights from GBD 2010 onward is likely contributing to the use of weights from earlier versions of the GBD estimates, especially in analyses which include the burden of congenital syphilis.

The main strength of this paper is its comprehensive overview of the use of health state utility and disability weights for economic evaluations of syphilis. This includes a comparative analysis of economic evaluations and primary sources. Limitations of this study include the omission of studies not published in English, though empirical evidence demonstrates little impact on systematic review conclusions [71, 72], and that we did not explicitly search the grey literature. We restricted our search for publications after January 2000, which may have resulted in missing some older studies. Overall, the quality assessment of economic evaluations was high – however, no studies presented a health economic analysis plan and distributional effects were seldom discussed. Another limitation is the exclusion of economic evaluations which did not explicitly provide a numerical weight for a health state utility value related to syphilis, noting that 42 out of 90 studies which were assessed for eligibility did not explicitly provide the numerical weights used in their analyses – in this way, we may be underestimating the interconnectedness of the literature and the reuse of the same weights by studies which do not have explicitly stated weights in their manuscripts but have still used them to calculate cost-effectiveness.

### Future research

We have uncovered a gap in the literature which deserves further research: the creation of validated and societally representative weights for both congenital and adult syphilis to help inform economic evaluations. Consensus is required firstly for the weights of standardised stages of syphilis and secondly, on the median durations over which to apply them. This ensures economic evaluations draw accurate conclusions which then inform policymaking. Without accurate weights or durations, researchers should use sensitivity analyses for DALY and QALY calculations. Research into the secondary or collateral effects of disease is ongoing. Uniquely, one study in our review considered the maternal perspective of sequelae of congenital syphilis [36]. Poorly-captured effects of syphilis on quality of life – such as the effects of stigma or the effect of a stillbirth on a mother or family – should be researched and incorporated for more grounded results [73]. As health equity rises in importance as a policymaking agenda, it is important to recognise that syphilis is a disease which disproportionately affects those from low-income countries [74]. Equity impact analysis as a tool may further influence syphilis health state utility values and lead to more equitable policy outcomes [75].

## Conclusion

Economic evaluations, which include syphilis utility or disability weights, are often recycling weights adapted from very different methods, primary sources, disease stage classifications and durations, which on further investigation, are based on limited evidence that is outdated and questionable in terms of its external validity. Aligning to recent updates for disability weights by the Global Burden of Disease initiative could be a pragmatic starting point to standardise. Given the complexity of syphilis and its wide variety of clinical health states, guidance is needed from the Global Burden of Disease team on how to correctly apply the disability weights by standardised clinical stages with consensus duration estimates. Until researchers use more accurate weights in economic evaluations, policymakers may be misinformed when considering the cost-effectiveness of syphilis programs.

### Supplementary Information

**Additional file 1.**

## Data Availability

All data generated or analysed during this study are included in this published article and its supplementary information files.
