# Supplementary material for: Ignored and undervalued in public health: a systematic review of health state utility values associated with syphilis infection
Source: Health Qual Life Outcomes. 2024 Feb 13;22:17. doi: 10.1186/s12955-024-02234-1 (PMC10863090; doi:10.1186/s12955-024-02234-1)
Supplement: Supplementary file 1 — Additional file 1. [file 12955_2024_2234_MOESM1_ESM.docx]

**Syphilis Systematic Supplementary Materials**

**Supplementary File S1: Methodology behind the development of the disability weights for the two captured primary sources**

*Primary study 1: Bennett (2000)*

Two economic evaluations used a condition specific utility weight of 0·74 for congenital syphilis derived from bacterial meningitis.(1, 2) The primary study for these was based on a valuation study of Canadian parents (n=94) presenting to a paediatric emergency department with a febrile child aged 3 to 36 months or had a sibling in that age range who were asked to value health states associated with meningitis. They performed an initial ranking of eight outcomes of bacteraemia using a visual-analogue scale then used a chained standard gamble to provide specific utility weights. Instead of a normal standard gamble in which subjects select a probability of certain death to avoid a health outcome, the chained standard gamble involved the subjects selecting the probability of perfect health against acquiring the next ranked outcome of bacteraemia as per their visual-analogue scale.(3) The outcome which was selected for use in syphilis economic evaluations was termed “meningitis with minor brain damage”, which had a mean utility weight of 0.7393 and a standard deviation of 0.29 and a median of 0.8681. It was ranked better than “meningitis with severe brain damage” and worse than “meningitis with deafness”. A later study, a cost-utility analysis of newborn screening strategies, adapted the value for “meningitis with mild brain damage” and applied it to a health state called “mild developmental delay”, which was the value cited in the economic evaluations.(4)

*Primary study 2: Castillo (2021)*

The second primary study developed its own utility weight by adapting the EuroQol-5 Dimensions (EQ-5D), a generic preference measure, and conducted interviews of two local populations in Chile, the first being 29 inmates infected with syphilis from a prison in Arica city, and the second being 67 patients from a sexually transmitted infection outpatient clinic in Santiago.(5) The utility weight for syphilis infection was calculated using a multi-attribute utility instrument by taking the mean value for the combined sample of the two populations (n=96), giving a final valuation of 0·737. (6)

**Supplementary File S2: Methodology behind the development of the 1990 Global Burden of Disease (GBD) disability weights**

The methodology behind the development of the 1990 GBD disability weights was based on a person trade-off approach. This involved a group of 8-12 health experts from all regions of the world to initially set the disability weights for 22 indicator conditions in seven classes, ranging from “vitiligo on the face” in Class I to “active psychosis” in Class VII. They were then asked how many people in a health state had an equal claim on a fixed healthcare budget as 1000 healthy people. If health experts believed that 8000 people had an equal claim on healthcare resources as 1000 healthy people, then the resulting disability weight for the condition would be 1 minus 1000 divided by 8000, or 0.875. These experts were also asked to make judgements on the social preferences for the length of times they would prefer to spend in specific health states. After the 22 indicator conditions have been weighted, then the remaining over 200 conditions were assigned across the seven classes.(7)

**Supplementary File S3: Methodology and expansion of updated weights for GBD from 2010 onwards**

From 2010 onward, the development of disability weights was significantly changed were made from the initial methodology from the 1990s. Firstly, there was a conceptual change from disability weights quantifying welfare loss to now quantifying health loss (and so the weighting of a condition such as blindness fell from 0.594 to 0.195, because respondents may not consider blind people to be sick in the common use of the term). Secondly, the methodology was changed from a person trade-off method to a discrete choice model. Thirdly, the pool of respondents was changed from expert panels of healthcare professionals to the general population.(8) For GBD 2010, there were 13902 household surveys held between five countries (Bangladesh, Indonesia, Peru, Tanzania, and the USA) and 16328 web-based surveys conducted.(8) For GBD 2013, 30660 further web-based surveys were held in four countries (Hungary, Italy, Netherlands, and Sweden).(9)

Instead of providing each health state with its own numerical disability weight value as was done previously (i.e. congenital syphilis was simply congenital syphilis), for publications from 2010 onwards, the GBD mapped unique sequelae (e.g. “mild early syphilis infection”) to a corresponding non-specific health state (e.g. “infectious disease, acute episode, mild”) which had an assigned disability weight. In 2010 and 2013 updates no syphilis sequelae were available. In the 2015 publication, three sequelae and health states associated with syphilis were introduced, and in the 2017 and 2019 publications, this was expanded to ten sequelae with their corresponding health states. The only difference between the 2017 and 2019 publications was the calculation of some combined disability weights which were not yet calculated in the 2017 publication. In the 2019 version, weights range from 0 for asymptomatic infection to 0·006 for “mild early syphilis infection” (health state: “infectious disease, acute episode, mild”), to 0·547 for “severe disfigurement, neurological problems, and cardiovascular complications due to adult tertiary syphilis” (combining the three health states: “Level 3 disfigurement”, “moderate motor plus cognitive impairments”, and “moderate infectious disease, acute episode”).(10-14)

**Supplementary Tables S1-7: Search Methodology**

Six databases were searched on January 7 2021. The search looked for mentions of syphilis, quality of life, measures of health state utility, economic evaluations, and economic models. The search limits were from 2000-current, humans, and English language. The search strategy was refined with the project team until the results retrieved reflected the scope of the project. The final Medline search was amended to run across the other databases. Note that funding for the EBM Reviews databases ceased at the end of 2015 and so coverage does not extend to the date of the search.

The databases searched were:

1. OvidSP MEDLINE and Epub Ahead of Print, In-Process & Other Non-Indexed Citations, Daily and Versions, 1946 to January 7, 2020
2. OvidSP Embase Classic + Embase, 1947 to January 7, 2020
3. OvidSP EBM Reviews – NHS Economic Evaluation Database, 1^st^ Quarter 2016
4. OvidSP EBM Reviews – Health Technology Assessment, 4^th^ Quarter 2016
5. OvidSP EBM Reviews – Database of Abstracts of Reviews of Effects, 1^st^ Quarter 2016
6. Web of Science Core Collection
   1. Science citation index expanded (1900- present)
   2. Social sciences citation index (1900 to present)
   3. Arts & humanities citation index (1975 to present)
   4. Conference proceedings citation index- Science (1990 to present)
   5. Conference proceedings citation index - Social science & humanities (1990 to present)
   6. Book citation index - science (2005 to present)
   7. Book citation index - Social sciences & humanities (2005 to present)
   8. Current chemical reactions (1985 to present)
   9. Index Chemicus (1993 to present).

Our initial search returned 3041 studies. Following title and abstract screening, 87 studies were selected for full-text review. In total, 19 economic evaluations met the inclusion criteria.(1, 2, 15-31) No primary studies were identified in the initial search, but two primary studies were added after hand searching reference lists.(3, 6) One of the studies added after hand searching was both an economic evaluation and a primary study.(6)

For the thirteen burden of disease studies, (10-14, 32-39) of these, two studies were found in the database search,(38, 39) and 11 studies were found by hand searching: seven GBD studies, (10-14, 32, 33) and four country-related landmark burden of disease studies.(34-37)

The search was updated on February 4, 2022, with the same terms, intending to search for studies published between the date of the initial search and February 4 2022. The updated search returned two additional economic evaluations.(40, 41)

The PRISMA flowchart for the updated search only is shown in Supplementary Figure S1.

**Supplementary Table S1: Databases searched**

| Database name | EndNote import order | Number of references before deduplication | Number of references after deduplication (removed) |
| --- | --- | --- | --- |
| Medline | 1 | 504 | 504 |
| Embase | 2 | 1246 | 978, (268) |
| NHS Economic Evaluation Database | 3 | 37 | 37 |
| Health Technology Assessment | 4 | 0 | 0 |
| Database of Abstracts of Reviews of Effects | 5 | 0 | 0 |
| Web of Science | 6 | 1254 | 961, (293) |
| **Total** |  | 3041 | 2480 |

**Supplementary Table S2: OvidSP Medline**

| Database name | Medline |
| --- | --- |
| Database platform | OvidSP |
| Dates of database coverage | 1946 to January 7 2021 |
| Date searched | 07/01/2021 |
| Searched by | PM |
| Number of hits | 504 |

1. exp Syphilis/ or Syphilis.mp. (34414)
2. exp Treponema pallidum/ or Treponema pallidum.mp. (5557)
3. exp "Quality of Life"/ or "Quality of Life".mp. or life quality.mp. or hql.mp. or qol.mp. or hrql.mp. or hrqol.mp. (310992)
4. (QALY$ or "Quality adjusted Life Year$").mp. or exp Quality-Adjusted Life Years/ (17657)
5. exp Health Status/ or (health status or health level).mp. (393367)
6. exp Health Status Indicators/ or Health Status Indicators.mp. (313500)
7. (utilit* or close utility analys* or health utilit* or disutility).mp. (178972)
8. exp Health Impact Assessment/ or Health Impact Assessment.mp. (1251)
9. (sf 36 or sf36 or "short form 36" or shortform 36 or sf thirtysix or "sf thirty six" or "short form thirty six" or "short form thirtysix" or "shortform thirty six" or "shortform thirtysix" or (sf 12 or sf12 or "short form 12" or shortform 12 or sf twelve or "short form twelve" or "shortform twelve") or (sf 8 or sf8 or "short form 8" or shortform 8 or sf eight or "short form eight" or "shortform eight") or (sf 6d or sf6d or "short form 6d" or shortform 6d or sf six or "short form six" or "shortform six")).tw. (28866)
10. (euroqol or euro qol or eq5d or eq 5d).mp. (9628)
11. (hui$ or health utilities index).mp. (6782)
12. (standard gamble or time trade off or tto or discrete choice experiment$ or discrete choice model$ or qualitative choice model$).mp. (3427)
13. exp Patient Preference/ or Patient Preference.mp. or (exp Patient Satisfaction/ or Patient Satisfaction.mp.) or preference$.mp. or valuation$.mp. (241616)
14. patient related outcome.mp. (231)
15. cost utility analys*.mp. (2641)
16. exp Cost-Benefit Analysis/ or Cost-Benefit Analys*.mp. or cost effective*.mp. (156359)
17. exp "Costs and Cost Analysis"/ (241491)
18. exp Models, Economic/ or (economic adj3 model$).mp. (17790)
19. markov$.mp. (22773)
20. economic evaluation$.mp. (10712)
21. 1 or 2 (35756)
22. or/3-20 (1394878)
23. 21 and 22 (805)
24. limit 23 to (english language and humans and yr="2000 -Current") (504)

**Supplementary Table S3: OvidSP Embase Classic + Embase**

| Database name | Embase Classic + Embase |
| --- | --- |
| Database platform | OvidSP |
| Dates of database coverage | 1946 to January 7 2021 |
| Date searched | 07/01/2021 |
| Searched by | PM |
| Number of hits | 1246 |

1. exp Syphilis/ or Syphilis.mp. (50494)
2. exp Treponema pallidum/ or Treponema pallidum.mp. (9790)
3. exp "Quality of Life"/ or "Quality of Life".mp. or life quality.mp. or hql.mp. or qol.mp. or hrql.mp. or hrqol.mp. (637945)
4. (QALY$ or "Quality adjusted Life Year$").mp. or exp Quality-Adjusted Life Years/ (34498)
5. exp Health Status/ or (health status or health level).mp. (291289)
6. exp Health Status Indicators/ or Health Status Indicators.mp. (33092)
7. (utilit* or close utility analys* or health utilit* or disutility).mp. (313482)
8. exp Health Impact Assessment/ or Health Impact Assessment.mp. (5858)
9. (sf 36 or sf36 or "short form 36" or shortform 36 or sf thirtysix or "sf thirty six" or "short form thirty six" or "short form thirtysix" or "shortform thirty six" or "shortform thirtysix" or (sf 12 or sf12 or "short form 12" or shortform 12 or sf twelve or "short form twelve" or "shortform twelve") or (sf 8 or sf8 or "short form 8" or shortform 8 or sf eight or "short form eight" or "shortform eight") or (sf 6d or sf6d or "short form 6d" or shortform 6d or sf six or "short form six" or "shortform six")).tw. (54570)
10. (euroqol or euro qol or eq5d or eq 5d).mp. (22617)
11. (hui$ or health utilities index).mp. (13415)
12. (standard gamble or time trade off or tto or discrete choice experiment$ or discrete choice model$ or qualitative choice model$).mp. (6264)
13. exp Patient Preference/ or Patient Preference.mp. or (exp Patient Satisfaction/ or Patient Satisfaction.mp.) or preference$.mp. or valuation$.mp. (376971)
14. patient related outcome.mp. (477)
15. cost utility analys*.mp. (11371)
16. exp Cost-Benefit Analysis/ or Cost-Benefit Analys*.mp. or cost effective*.mp. (329225)
17. exp "Costs and Cost Analysis"/ (363423)
18. exp Models, Economic/ or (economic adj3 model$).mp. (9156)
19. markov$.mp. (37242)
20. economic evaluation$.mp. (26537)
21. 1 or 2 (53297)
22. or/3-20 (2094046)
23. 21 and 22 (1706)
24. limit 23 to (english language and humans and yr="2000 -Current") (1246)

**Supplementary Table S4: OvidSP NHS Economic Evaluation Database**

| Database name | NHS Economic Evaluation Database |
| --- | --- |
| Database platform | OvidSP |
| Dates of database coverage | 1^st^ Quarter 2016 |
| Date searched | 07/01/2021 |
| Searched by | PM |
| Number of hits | 37 |

1. exp Syphilis/ or Syphilis.mp. (42)
2. exp Treponema pallidum/ or Treponema pallidum.mp. (8)
3. exp "Quality of Life"/ or "Quality of Life".mp. or life quality.mp. or hql.mp. or qol.mp. or hrql.mp. or hrqol.mp. (6244)
4. (QALY$ or "Quality adjusted Life Year$").mp. or exp Quality-Adjusted Life Years/ (4752)
5. exp Health Status/ or (health status or health level).mp. (540)
6. exp Health Status Indicators/ or Health Status Indicators.mp. (512)
7. (utilit* or close utility analys* or health utilit* or disutility).mp. (4588)
8. exp Health Impact Assessment/ or Health Impact Assessment.mp. (1)
9. (sf 36 or sf36 or "short form 36" or shortform 36 or sf thirtysix or "sf thirty six" or "short form thirty six" or "short form thirtysix" or "shortform thirty six" or "shortform thirtysix" or (sf 12 or sf12 or "short form 12" or shortform 12 or sf twelve or "short form twelve" or "shortform twelve") or (sf 8 or sf8 or "short form 8" or shortform 8 or sf eight or "short form eight" or "shortform eight") or (sf 6d or sf6d or "short form 6d" or shortform 6d or sf six or "short form six" or "shortform six")).tw. (300)
10. (euroqol or euro qol or eq5d or eq 5d).mp. (731)
11. (hui$ or health utilities index).mp. (114)
12. (standard gamble or time trade off or tto or discrete choice experiment$ or discrete choice model$ or qualitative choice model$).mp. (498)
13. exp Patient Preference/ or Patient Preference.mp. or (exp Patient Satisfaction/ or Patient Satisfaction.mp.) or preference$.mp. or valuation$.mp. (3861)
14. patient related outcome.mp. (0)
15. cost utility analys*.mp. (3136)
16. exp Cost-Benefit Analysis/ or Cost-Benefit Analys*.mp. or cost effective*.mp. (14957)
17. exp "Costs and Cost Analysis"/ (14740)
18. exp Models, Economic/ or (economic adj3 model$).mp. (1833)
19. markov$.mp. (2735)
20. economic evaluation$.mp. (17190)
21. 1 or 2 (45)
22. or/3-20 (17397)
23. 21 and 22 (45)
24. limit 23 to (english language and humans and yr="2000 -Current") (37)

**Supplementary Table S5: OvidSP Health Technology Assessment**

| Database name | Health Technology Assessment |
| --- | --- |
| Database platform | OvidSP |
| Dates of database coverage | 4^th^ Quarter 2016 |
| Date searched | 07/01/2021 |
| Searched by | PM |
| Number of hits | 0 |

1. exp Syphilis/ or Syphilis.mp. (1)
2. exp Treponema pallidum/ or Treponema pallidum.mp. (1)
3. exp "Quality of Life"/ or "Quality of Life".mp. or life quality.mp. or hql.mp. or qol.mp. or hrql.mp. or hrqol.mp. (787)
4. (QALY$ or "Quality adjusted Life Year$").mp. or exp Quality-Adjusted Life Years/ (214)
5. exp Health Status/ or (health status or health level).mp. (71)
6. exp Health Status Indicators/ or Health Status Indicators.mp. (29)
7. (utilit* or close utility analys* or health utilit* or disutility).mp. (239)
8. exp Health Impact Assessment/ or Health Impact Assessment.mp. (7)
9. (sf 36 or sf36 or "short form 36" or shortform 36 or sf thirtysix or "sf thirty six" or "short form thirty six" or "short form thirtysix" or "shortform thirty six" or "shortform thirtysix" or (sf 12 or sf12 or "short form 12" or shortform 12 or sf twelve or "short form twelve" or "shortform twelve") or (sf 8 or sf8 or "short form 8" or shortform 8 or sf eight or "short form eight" or "shortform eight") or (sf 6d or sf6d or "short form 6d" or shortform 6d or sf six or "short form six" or "shortform six")).tw. (13)
10. (euroqol or euro qol or eq5d or eq 5d).mp. (15)
11. (hui$ or health utilities index).mp. (1)
12. (standard gamble or time trade off or tto or discrete choice experiment$ or discrete choice model$ or qualitative choice model$).mp. (7)
13. exp Patient Preference/ or Patient Preference.mp. or (exp Patient Satisfaction/ or Patient Satisfaction.mp.) or preference$.mp. or valuation$.mp. (257)
14. patient related outcome.mp. (0)
15. cost utility analys*.mp. (23)
16. exp Cost-Benefit Analysis/ or Cost-Benefit Analys*.mp. or cost effective*.mp. (1946)
17. exp "Costs and Cost Analysis"/ (1198)
18. exp Models, Economic/ or (economic adj3 model$).mp. (136)
19. markov$.mp. (11)
20. economic evaluation$.mp. (499)
21. 1 or 2 (1)
22. or/3-20 (3343)
23. 21 and 22 (0)
24. limit 23 to (english language and humans and yr="2000 -Current") (0)

**Supplementary Table S6: OvidSP Database of Abstracts of Reviews of Effects**

| Database name | Database of Abstracts of Reviews of Effects |
| --- | --- |
| Database platform | OvidSP |
| Dates of database coverage | 1^st^ Quarter 2016 |
| Date searched | 07/01/2021 |
| Searched by | PM |
| Number of hits | 0 |

1. syphilis.mp. (16)
2. Treponema pallidum.mp. (2)
3. ("Quality of Life" or life quality or hql or qol or hrql or hrqol).mp. (2214)
4. (QALY$ or "Quality adjusted Life Year$").mp. (156)
5. (health status or health level).mp. (380)
6. Health Status Indicators.mp. (44)
7. (utilit* or close utility analys* or health utilit* or disutility).mp. (330)
8. Health Impact Assessment.mp. (1)
9. (sf 36 or sf36 or "short form 36" or shortform 36 or sf thirtysix or "sf thirty six" or "short form thirty six" or "short form thirtysix" or "shortform thirty six" or "shortform thirtysix" or (sf 12 or sf12 or "short form 12" or shortform 12 or sf twelve or "short form twelve" or "shortform twelve") or (sf 8 or sf8 or "short form 8" or shortform 8 or sf eight or "short form eight" or "shortform eight") or (sf 6d or sf6d or "short form 6d" or shortform 6d or sf six or "short form six" or "shortform six")).tw. (125)
10. (euroqol or euro qol or eq5d or eq 5d).mp. (22)
11. (hui$ or health utilities index).mp. (1)
12. (standard gamble or time trade off or tto or discrete choice experiment$ or discrete choice model$ or qualitative choice model$).mp. (2)
13. (Patient Preference or Patient Satisfaction or preference$ or valuation$).mp. (964)
14. patient related outcome.mp. (4)
15. cost utility analys*.mp. (33)
16. (Cost-Benefit Analys* or cost effective*).mp. (1626)
17. (economic adj3 model*).mp. (93)
18. markov$.mp. (73)
19. economic evaluation$.mp. (335)
20. 1 or 2 (16)
21. or/3-19 (4633)
22. 20 and 21 (0)

**Supplementary Table S7: Web of Science**

| Database name | Web of Science Core Collection |
| --- | --- |
| Database platform | Web of Science |
| Dates of database coverage | No list available |
| Date searched | 07/01/2021 |
| Searched by | PM |
| Number of hits | 1254 |

1. ALL=(syphilis or treponema pallidum) (32180)
2. TS=(quality of life or quality adjusted life year* or QALY*) (569560)
3. TS=(health status or health state* or health status indicator*) (568999)
4. TS=(utility* or health utility* or disutility) (431196)
5. TS=(health impact assessment*) (58937)
6. TS=((sf 36 or sf36 or "short form 36" or shortform 36 or sf thirtysix or "sf thirty six" or "short form thirty six" or "short form thirtysix" or "shortform thirty six" or "shortform thirtysix" or (sf 12 or sf12 or "short form 12" or shortform 12 or sf twelve or "short form twelve" or "shortform twelve") or (sf 8 or sf8 or "short form 8" or shortform 8 or sf eight or "short form eight" or "shortform eight") or (sf 6d or sf6d or "short form 6d" or shortform 6d or sf six or "short form six" or "shortform six")) (47977)
7. TS=(euroqol or euro qol or eq5d or eq 5d or hui$ or health utilities index or standard gamble or time trade off or tto or discrete choice experiment$ or discrete choice model$ or qualitative choice model$) (69219)
8. TS=(patient preference$ or patient satisfaction or preference$ or valuation$ or patient related outcome$) (752663)
9. TS=(cost utility analys?s or cost benefit analys?s or cost analys?s or economic evaluation$) (520147)
10. TS=(economic model* or economic NEAR/3 model* or markov$) (402352)
11. #2 OR #3 OR #4 OR #5 OR #6 OR #7 OR #8 OR #9 OR #10 (2955761)
12. (#1 AND #11) AND language: (English) AND Timespan=2000-2021 (1254)

**Supplementary Tables S8-10: Quality Assessment Tables**

**Supplementary Table S8: Consolidated Health Economic Evaluation Reporting Standards (CHEERS) 2022 for economic evaluations(42)**

| CHEERS 2022 | 1 | 2 | 3 | 4 | 5 | 6 | 7 | 8 | 9 | 10 | 11 | 12 | 13 | 14 | 15 | 16 | 17 | 18 | 19 | 20 | 21 | 22 | 23 | 24 | 25 | 26 | 27 | 28 |
| --- | --- | --- | --- | --- | --- | --- | --- | --- | --- | --- | --- | --- | --- | --- | --- | --- | --- | --- | --- | --- | --- | --- | --- | --- | --- | --- | --- | --- |
| Bristow (15) | Y | Y | Y | N | Y | Y | Y | N | Y | Y | Y | N | Y | Y | Y | Y | Y | N | N | Y | N | Y | Y | Y | N | Y | Y | Y |
| Castillo (6) | Y | Y | Y | N | Y | Y | Y | Y | Y | Y | Y | Y | Y | Y | N | Y | Y | N | N | Y | N | Y | Y | Y | N | Y | Y | Y |
| Custer (41) | Y | Y | Y | N | Y | Y | Y | Y | N | Y | Y | Y | Y | Y | Y | Y | Y | Y | N | N | Y | Y | Y | Y | N | Y | Y | Y |
| Eaton (29) | Y | N | Y | N | Y | Y | Y | Y | Y | N | N | Y | Y | Y | Y | N | Y | N | N | N | N | Y | Y | Y | N | Y | Y | Y |
| Hersh (1) | Y | Y | Y | N | Y | Y | Y | Y | Y | Y | Y | Y | Y | Y | Y | Y | Y | N | N | N | N | Y | Y | Y | N | Y | N | Y |
| Hong (16) | Y | Y | Y | N | Y | Y | Y | N | Y | Y | Y | Y | Y | Y | Y | N | Y | N | N | N | N | N | Y | N | N | Y | Y | N |
| Huntington (2) | Y | Y | Y | N | Y | Y | Y | Y | Y | Y | Y | Y | Y | Y | Y | Y | Y | Y | N | Y | Y | Y | Y | Y | Y | Y | Y | Y |
| Jayawardena (17) | N | Y | Y | N | Y | Y | Y | N | Y | N | Y | Y | Y | Y | N | Y | Y | Y | Y | N | N | Y | Y | Y | Y | Y | N | N |
| Kahn (18) | Y | Y | Y | N | Y | Y | Y | Y | Y | Y | Y | Y | Y | Y | Y | Y | Y | Y | N | N | N | Y | Y | Y | N | Y | Y | Y |
| Kuznik (19) | Y | Y | Y | N | Y | Y | Y | Y | Y | Y | Y | N | Y | Y | Y | Y | Y | N | N | N | N | Y | Y | Y | N | Y | Y | Y |
| Kuznik (20) | Y | Y | Y | N | Y | Y | Y | Y | Y | Y | Y | N | Y | Y | Y | Y | Y | Y | N | Y | N | Y | Y | Y | N | Y | Y | Y |
| Larson (21) | Y | Y | Y | N | Y | Y | Y | Y | Y | Y | Y | N | Y | Y | Y | Y | Y | N | N | Y | N | Y | Y | Y | N | Y | Y | Y |
| Owusu-Edusei (22) | Y | Y | Y | N | Y | Y | Y | Y | Y | Y | Y | N | Y | Y | Y | Y | Y | N | N | N | N | Y | Y | Y | N | Y | Y | N |
| Owusu-Edusei (23) | Y | Y | Y | N | Y | Y | Y | Y | Y | Y | Y | N | Y | Y | Y | Y | Y | N | N | N | N | Y | Y | Y | N | Y | Y | Y |
| Rodriguez (24) | Y | Y | Y | N | Y | Y | Y | Y | Y | Y | Y | N | Y | Y | Y | Y | Y | Y | N | Y | N | Y | Y | Y | N | Y | Y | Y |
| Romero (25) | Y | Y | Y | N | Y | Y | Y | Y | Y | Y | Y | Y | Y | Y | Y | Y | Y | Y | N | Y | N | Y | Y | Y | N | Y | Y | N |
| Russell (40) | Y | Y | Y | N | Y | Y | Y | Y | Y | Y | Y | Y | Y | Y | Y | Y | Y | N | N | Y | N | Y | Y | Y | N | Y | Y | Y |
| Schackman (26) | Y | Y | Y | N | Y | Y | Y | Y | Y | Y | Y | Y | Y | Y | Y | Y | Y | Y | N | N | Y | Y | Y | Y | N | Y | Y | Y |
| Sujikerbuijk (30) | N | Y | Y | N | Y | Y | Y | Y | Y | Y | Y | Y | Y | Y | Y | Y | N | Y | N | N | N | Y | Y | Y | N | Y | N | Y |
| Terris-Prestholt (27) | Y | Y | Y | N | Y | Y | Y | N | Y | Y | Y | Y | Y | Y | Y | N | Y | Y | N | Y | N | Y | Y | Y | N | Y | Y | Y |
| Terris-Prestholt (28) | Y | Y | Y | N | Y | Y | Y | Y | Y | Y | Y | Y | Y | Y | Y | Y | Y | N | N | N | N | Y | Y | Y | N | Y | N | N |
| Tuite (31) | Y | Y | Y | N | Y | Y | Y | Y | Y | Y | Y | Y | Y | Y | Y | Y | Y | Y | Y | Y | N | Y | Y | Y | N | Y | Y | Y |

Checklist criteria:

Title

1. Title - Identify the study as an economic evaluation and specify the interventions being compared.

Abstract

1. Abstract - Provide a structured summary that highlights context, key methods, results and alternative analyses.

Introduction

1. Background and objectives - Give the context for the study, the study question and its practical relevance for decision making in policy or practice.

Methods

1. Health economic analysis plan - Indicate whether a health economic analysis plan was developed and where available.
2. Study population - Describe characteristics of the study population (such as age range, demographics, socioeconomic, or clinical characteristics).
3. Setting and location - Provide relevant contextual information that may influence findings.
4. Comparators - Describe the interventions or strategies being compared and why chosen.
5. Perspective - State the perspective(s) adopted by the study and why chosen.
6. Time horizon - State the time horizon for the study and why appropriate.
7. Discount rate - Report the discount rate(s) and reason chosen.
8. Selection of outcomes - Describe what outcomes were used as the measure(s) of benefit(s) and harm(s).
9. Measurement of outcomes - Describe how outcomes used to capture benefit(s) and harm(s) were measured.
10. Valuation of outcomes - Describe the population and methods used to measure and value outcomes.
11. Measurement and valuation of resources and costs - Describe how costs were valued.
12. Currency, price date, and conversion - Report the dates of the estimated resource quantities and unit costs, plus the currency and year of conversion.
13. Rationale and description of model - If modelling is used, describe in detail and why used. Report if the model is publicly available and where it can be accessed.
14. Analytics and assumptions - Describe any methods for analysing or statistically transforming data, any extrapolation methods, and approaches for validating any model used.
15. Characterising heterogeneity - Describe any methods used for estimating how the results of the study vary for sub-groups.
16. Characterising distributional effects - Describe how impacts are distributed across different individuals or adjustments made to reflect priority populations.
17. Characterising uncertainty - Describe methods to characterize any sources of uncertainty in the analysis.
18. Approach to engagement with patients and others affected by the study - Describe any approaches to engage patients or service recipients, the general public, communities, or stakeholders (e.g., clinicians or payers) in the design of the study.

Results

1. Study parameters - Report all analytic inputs (e.g., values, ranges, references) including uncertainty or distributional assumptions.
2. Summary of main results - Report the mean values for the main categories of costs and outcomes of interest and summarise them in the most appropriate overall measure.
3. Effect of uncertainty - Describe how uncertainty about analytic judgments, inputs, or projections affect findings. Report the effect of choice of discount rate and time horizon, if applicable.
4. Effect of engagement with patients and others affected by the study - Report on any difference patient/service recipient, general public, community, or stakeholder involvement made to the approach or findings of the study.

Discussion

1. Study findings, limitations, generalisability, and current knowledge - Report key findings, limitations, ethical or equity considerations not captured, and how these could impact patients, policy, or practice.

Other relevant information

1. Source of funding - Describe how the study was funded and any role of the funder in the identification, design, conduct, and reporting of the analysis
2. Conflicts of interest - Report authors conflicts of interest according to journal or International Committee of Medical Journal Editors requirements.

**Supplementary Table S9: Consensus Health Economic Criteria (CHEC) List for economic evaluations(43)**

| CHEC | 1 | 2 | 3 | 4 | 5 | 6 | 7 | 8 | 9 | 10 | 11 | 12 | 13 | 14 | 15 | 16 | 17 | 18 | 19 |
| --- | --- | --- | --- | --- | --- | --- | --- | --- | --- | --- | --- | --- | --- | --- | --- | --- | --- | --- | --- |
| Bristow (15) | Y | Y | Y | Y | Y | N | Y | Y | Y | Y | N | Y | Y | Y | Y | Y | N | Y | N |
| Castillo (6) | Y | Y | Y | Y | Y | Y | Y | Y | N | Y | Y | Y | Y | Y | Y | Y | Y | Y | N |
| Custer (41) | Y | Y | Y | Y | Y | Y | Y | Y | Y | Y | Y | Y | Y | Y | Y | Y | Y | Y | N |
| Eaton (29) | Y | Y | Y | Y | Y | Y | N | Y | Y | N | Y | Y | Y | N | N | Y | N | Y | N |
| Hersh (1) | Y | Y | Y | Y | Y | Y | Y | Y | Y | Y | Y | Y | Y | Y | N | Y | N | N | N |
| Hong (16) | Y | Y | Y | Y | Y | N | Y | Y | Y | Y | Y | Y | N | Y | N | Y | N | N | N |
| Huntington (2) | Y | Y | Y | Y | Y | Y | Y | Y | Y | Y | Y | Y | Y | Y | Y | Y | Y | Y | N |
| Jayawardena (17) | Y | Y | Y | Y | Y | N | Y | Y | N | Y | Y | Y | Y | N | N | Y | Y | N | Y |
| Kahn (18) | Y | Y | Y | Y | Y | Y | Y | Y | Y | Y | Y | Y | Y | Y | N | Y | Y | N | N |
| Kuznik (19) | Y | Y | Y | Y | Y | Y | Y | Y | Y | Y | N | Y | Y | Y | N | Y | Y | N | N |
| Kuznik (20) | Y | Y | Y | Y | Y | Y | Y | Y | Y | Y | N | Y | Y | Y | Y | Y | Y | N | N |
| Larson (21) | Y | Y | Y | Y | Y | Y | Y | Y | Y | Y | N | Y | N | Y | Y | Y | Y | Y | N |
| Owusu-Edusei (22) | Y | Y | Y | Y | Y | Y | Y | Y | Y | Y | N | Y | Y | Y | N | Y | N | N | N |
| Owusu-Edusei (23) | Y | Y | Y | Y | Y | Y | Y | Y | Y | Y | N | Y | Y | Y | N | Y | Y | Y | N |
| Rodriguez (24) | Y | Y | Y | Y | Y | Y | Y | Y | Y | Y | N | Y | Y | Y | Y | Y | Y | Y | N |
| Romero (25) | Y | Y | Y | Y | Y | Y | Y | Y | Y | Y | Y | Y | Y | Y | Y | Y | Y | Y | N |
| Russell (40) | Y | Y | Y | Y | Y | Y | Y | Y | Y | Y | Y | Y | Y | Y | Y | Y | Y | Y | N |
| Schackman (26) | Y | Y | Y | Y | Y | Y | Y | Y | Y | Y | Y | Y | Y | Y | N | Y | Y | Y | N |
| Sujikerbuijk (30) | Y | Y | Y | Y | Y | Y | Y | Y | Y | Y | Y | Y | Y | Y | N | Y | N | Y | N |
| Terris-Prestholt (27) | Y | Y | Y | Y | Y | N | Y | Y | Y | Y | Y | Y | Y | Y | Y | Y | Y | Y | N |
| Terris-Prestholt (28) | Y | Y | Y | Y | Y | Y | Y | Y | Y | Y | Y | Y | Y | Y | N | Y | Y | N | N |
| Tuite (31) | Y | Y | Y | Y | Y | Y | Y | Y | Y | Y | Y | Y | Y | Y | Y | Y | Y | Y | N |

Checklist criteria:

1. Is the study population clearly described?
2. Are competing alternatives clearly described?
3. Is a well-defined research question posed in answerable form?
4. Is the economic study design appropriate to the stated objective?
5. Is the chosen time horizon appropriate in order to include relevant costs and consequences?
6. Is the actual perspective chosen appropriate?
7. Are all important and relevant costs for each alternative identified?
8. Are all costs measured appropriately in physical units?
9. Are costs valued appropriately?
10. Are all important and relevant outcomes for each alternative identified?
11. Are all outcomes measured appropriately?
12. Are outcomes valued appropriately?
13. Is an incremental analysis of costs and outcomes of alternatives performed?
14. Are all future costs and outcomes discounted appropriately?
15. Are all important variables, whose values are uncertain, appropriately subjected to sensitivity analysis?
16. Do the conclusions follow from the data reported?
17. Does the study discuss the generalizability of the results to other settings and patient/client groups?
18. Does the article indicate that there is no potential conflict of interest of study researcher(s) and funder(s)?
19. Are ethical and distributional issues discussed appropriately?

**Supplementary Table S10: Picot et al. Checklist for primary studies(44)**

|  | Bennett 2000 (3) | Castillo 2021 (6) |
| --- | --- | --- |
| 1 | Yes, primary outcome | Yes, secondary outcome |
| 2 | Yes | Yes |
| 3 | Yes | Yes |
| 4 | Yes | Yes |
| 5 | Yes | No |
| 6 | Yes | No |
| 7 | Yes | Yes |
| 8 | Yes | No |
| 9 | Yes | No |
| 10 | Yes, reported; no, unlikely to threaten validity | No, not reported; unclear if threatens validity |
| 11 | N/A | No |
| 12 | No, not reported; unclear if threatens validity | No, not reported; unclear if threatens validity |
| 13 | Yes | Yes |
| 14 | Yes | Yes |
| 15 | Yes | Yes |

Checklist criteria:

Conceptual

1. Study objectives - Were the objectives of the study clearly stated? HRQoL primary or secondary outcome?
2. HRQoL instrument - Was a reason provided to justify the HRQoL instrument selected? Was a validated tool used to assess QoL?

Methodology

1. Study design - Was the design of the study clearly described? (e.g. cohort, cross-sectional, survey)
2. Respondent selection and recruitment - Was the sampling method for recruitment of participants adequately described?
3. Inclusion/exclusion criteria - Are inclusion/exclusion criteria clearly described? Do these exclude any individuals that might be relevant (e.g. very elderly aged > 80 years old)?
4. Participant characteristics - Were characteristics of participants clearly described (demographics and clinical variables)?
5. Sample size - Was the sample size used appropriately justified?
6. Instrument administration - Is it reported who and/or in which clinical setting the instrument was administered?
7. Timing of assessments - Is the timing of assessments reported? (e.g. baseline and/or at follow-up or after treatment)

Results

1. Response rates to instrument used - Are response rates reported and if so, are the rates likely to be a threat to validity?
2. Loss to follow-up - Is the loss to follow-up reported and are reasons given? Are these likely to threaten the validity of results (e.g. characteristics of non-responders different to responders)?
3. Missing data - Are the levels of missing data reported? How are they dealt with? Could this threaten the validity of results?
4. Statistical analysis - Were appropriate statistical methods used?

Interpretation

1. Study findings - Were the key findings of the study clearly stated?
2. Study limitations - Were limitations of the study clearly described?

**Supplementary Table S11: Overview of health economic evaluation studies and burden of disease studies related to syphilis infection and primary studies evaluating syphilis-related quality of life.**

| **Economic evaluation studies** | **Total (*N*=22)** |
| --- | --- |
| *World Bank Income Classification 2021 (45)* | **n (%**^†^**)** |
| Low | 2 (9%) |
| Lower-middle | 3 (14%) |
| Upper-middle | 3 (14%) |
| High | 8 (36%) |
| Mixed | 6 (27%) |
| *Populations* |  |
| Pregnant women and neonates | 15 (68%) |
| Patients receiving blood transfusions | 2 (9%) |
| Blood donors | 1 (5%) |
| Prisoners | 1 (5%) |
| People living with HIV | 1 (5%) |
| STI clinic attendees | 1 (5%) |
| MSM living with HIV | 1 (5%) |
| *Publication year* |  |
| 2010 or before | 4 (18%) |
| 2011-2022 | 18 (82%) |
| **Primary studies** | **Total (*N*=2)** |
| *Country income level** | **n (%**^†^**)** |
| High | 2 (100%) |
| *Populations* |  |
| Adults | 2 (100%) |
| *Publication year* |  |
| 2010 or before | 1 (50%) |
| 2011-2022 | 1 (50%) |
| **Burden of disease studies** | **Total (*N*=13)** |
| *Country income level** | **n (%**^†^**)** |
| Global Burden of Disease | 7 (54%) |
| Upper-middle | 1 (8%) |
| High | 4 (31%) |
| Mixed | 1 (8%) |
| *Publication year* |  |
| 2010 or before | 6 (46%) |
| 2011-2022 | 7 (54%) |

HIV= human immunodeficiency virus, STI = sexually transmitted disease, MSM = men who have sex with men

^†^ Percentages may not add up to 100 due to rounding.

**Supplementary Figure S1: PRISMA Flowchart for Updated Search**

**Identification of studies via other methods**

**Identification of studies via databases and registers**

Records identified from:

Citation searching (n = 1)

Records removed *before screening*:

Duplicate records removed (n = 21)

Records identified from:

Databases (n = 360)

**Identification**

Records excluded

(n = 333)

Records screened

(n = 339)

Reports not retrieved

(n = 0)

Reports sought for retrieval

(n = 1)

Reports not retrieved

(n = 0)

Reports sought for retrieval

(n = 6)

**Screening**

Reports excluded:

Protocol (n=1)

No values in study (n = 4)

Reports assessed for eligibility

(n = 6)

Reports excluded:

(n = 0)

Reports assessed for eligibility

(n = 1)

**Included**

Studies included in review

(n = 2)

**References**

1. Hersh AR, Megli CJ, Caughey AB. Repeat Screening for Syphilis in the Third Trimester of Pregnancy: A Cost-Effectiveness Analysis. Obstetrics & Gynecology. 2018;132(3):699-707.

2. Huntington S, Weston G, Seedat F, Marshall J, Bailey H, Tebruegge M, et al. Repeat screening for syphilis in pregnancy as an alternative screening strategy in the UK: A cost-effectiveness analysis. BMJ Open. 2020;10 (11) (no pagination)(e038505).

3. Bennett JE, Sumner W, Downs SM, Jaffe DM. Parents' Utilities for Outcomes of Occult Bacteremia. Archives of Pediatrics and Adolescent Medicine. 2000;154(1):43-8.

4. Carroll AE, Downs SM. Comprehensive cost-utility analysis of newborn screening strategies. Pediatrics. 2006;117(5):S287-S95.

5. Ferrari M, Sanhueza XA, González C, Normandin A, Becerra ID, Rebeco MBDG, Zamora MF. La carga oculta de sífilis en personas privadas de libertad: medición de prevalencia con test rápido en cárcel de Arica. Medwave. 2016;16:6654-.

6. Castillo-Laborde C, Najera-De Ferrari M, Gajardo P, Aguirre P, Ramirez H, Ramirez D, et al. Modelling the effect of syphilis screening at the entry point in chilean prisons. Value in Health. 2017;20 (9):A869-A70.

7. Murray CJL, Lopez AD, Harvard School of Public H, World Health O, World B. The global burden of disease : a comprehensive assessment of mortality and disability from diseases, injuries, and risk factors in 1990 and projected to 2020. Cambridge, MA: Cambridge, MA : Published by the Harvard School of Public Health on behalf of the World Health Organization and the World Bank

Distributed by Harvard University Press; 1996.

8. Salomon JAP, Vos TP, Hogan DRP, Gagnon MMS, Naghavi MP, Mokdad AP, et al. Common values in assessing health outcomes from disease and injury: disability weights measurement study for the Global Burden of Disease Study 2010. The Lancet. 2012;380(9859):2129-43.

9. Salomon JAP, Haagsma JAP, Davis AP, de Noordhout CMMD, Polinder SP, Havelaar AHP, et al. Disability weights for the Global Burden of Disease 2013 study. Lancet Glob Health. 2015;3(11):e712-e23.

10. Vos TP, Flaxman ADP, Abdalla SM, AlMazroa MAMD, Alvarado MBA, Andrews KGMPH, et al. Disability-adjusted life years (DALYs) for 291 diseases and injuries in 21 regions, 1990–2010: a systematic analysis for the Global Burden of Disease Study 2010. The Lancet. 2012;380(9859):2197-223.

11. Naghavi M, Wang H, Lozano R, Davis A, Liang X, Zhou M, et al. Global, regional, and national age–sex specific all-cause and cause-specific mortality for 240 causes of death, 1990–2013: a systematic analysis for the Global Burden of Disease Study 2013. The Lancet. 2015;385(9963):117-71.

12. Wang H, Naghavi M, Allen C, Barber RM, Bhutta Z, Carter A, et al. Global, regional, and national life expectancy, all-cause mortality, and cause-specific mortality for 249 causes of death, 1980–2015: a systematic analysis for the Global Burden of Disease Study 2015. The Lancet. 2016;388(10053):1459-544.

13. James SLG, Abate D, Abate KH, Abay SM, Abbafati C, Abbasi N, et al. Global, regional, and national incidence, prevalence, and years lived with disability for 354 diseases and injuries for 195 countries and territories, 1990-2017: a systematic analysis for the Global Burden of Disease Study 2017. The Lancet. 2018;392(10159):1789-858.

14. Vos T, Lim SS, Abbafati C, Abbas KM, Abbasi M, Abbasifard M, et al. Global burden of 369 diseases and injuries in 204 countries and territories, 1990–2019: a systematic analysis for the Global Burden of Disease Study 2019. The Lancet. 2020;396(10258):1204-22.

15. Bristow CC, Larson E, Anderson LJ, Klausner JD. Cost-effectiveness of HIV and syphilis antenatal screening: a modelling study. Sexually Transmitted Infections. 2016;92(5):340-6.

16. Hong FC, Liu JB, Feng TJ, Liu XL, Pan P, Zhou H, et al. Congenital syphilis: an economic evaluation of a prevention program in China. Sexually Transmitted Diseases. 2010;37(1):26-31.

17. Jayawardena T, Hoad V, Styles C, Seed C, Bentley P, Clifford V, et al. Modelling the risk of transfusion-transmitted syphilis: a reconsideration of blood donation testing strategies. Vox Sanguinis. 2019;114(2):107-16.

18. Kahn JG, Jiwani A, Gomez GB, Hawkes SJ, Chesson HW, Broutet N, et al. The cost and cost-effectiveness of scaling up screening and treatment of syphilis in pregnancy: a model. PLoS ONE. 2014;9(1):e87510.

19. Kuznik A, Lamorde M, Nyabigambo A, Manabe YC. Antenatal syphilis screening using point-of-care testing in Sub-Saharan African countries: a cost-effectiveness analysis. PLoS Med. 2013;10(11):e1001545.

20. Kuznik A, Muhumuza C, Komakech H, Marques EMR, Lamorde M. Antenatal syphilis screening using point-of- care testing in low- and middle-income countries in Asia and Latin America: A cost- effectiveness analysis. PLoS ONE. 2015;10 (5) (no pagination)(e0127379).

21. Larson BA, Lembela-Bwalya D, Bonawitz R, Hammond EE, Thea DM, Herlihy J. Finding a needle in the haystack: the costs and cost-effectiveness of syphilis diagnosis and treatment during pregnancy to prevent congenital syphilis in Kalomo District of Zambia. PLoS ONE. 2014;9(12):e113868.

22. Owusu-Edusei K, Jr., Gift TL, Ballard RC. Cost-effectiveness of a dual non-treponemal/treponemal syphilis point-of-care test to prevent adverse pregnancy outcomes in sub-Saharan Africa. Sexually Transmitted Diseases. 2011;38(11):997-1003.

23. Owusu-Edusei K, Jr., Tao G, Gift TL, Wang A, Wang L, Tun Y, et al. Cost-effectiveness of integrated routine offering of prenatal HIV and syphilis screening in China. Sexually Transmitted Diseases. 2014;41(2):103-10.

24. Rodriguez PJ, Roberts DA, Meisner J, Sharma M, Owiredu MN, Gomez B, et al. Cost-effectiveness of dual maternal HIV and syphilis testing strategies in high and low HIV prevalence countries: a modelling study. The Lancet Global Health. 2021;9(1):e61-e71.

25. Romero CP, Marinho DS, Castro R, de Aguiar Pereira CC, Silva E, Caetano R, et al. Cost-Effectiveness Analysis of Point-of-Care Rapid Testing Versus Laboratory-Based Testing for Antenatal Screening of Syphilis in Brazil. Value in Health Regional Issues. 2020;23:61-9.

26. Schackman BR, Neukermans CP, Fontain SN, Nolte C, Joseph P, Pape JW, Fitzgerald DW. Cost-effectiveness of rapid syphilis screening in prenatal HIV testing programs in Haiti. PLoS Medicine. 2007;4(5):e183.

27. Terris-Prestholt F, Watson-Jones D, Mugeye K, Kumaranayake L, Ndeki L, Weiss H, et al. Is antenatal syphilis screening still cost effective in sub-Saharan Africa? Sex Transm Infect. 2003;79(5):375-81.

28. Terris-Prestholt F, Vickerman P, Torres-Rueda S, Santesso N, Sweeney S, Mallma P, et al. The cost-effectiveness of 10 antenatal syphilis screening and treatment approaches in Peru, Tanzania, and Zambia. International Journal of Gynaecology & Obstetrics. 2015;130 Suppl 1:S73-80.

29. Eaton EF, Joe W, Kilgore ML, Muzny CA. Reverse syphilis screening algorithm fails to demonstrate cost effectiveness in persons living with HIV. International Journal of STD & AIDS. 2018;29(6):563-7.

30. Suijkerbuijk AWM, Over EAB, van Aar F, Gotz HM, van Benthem BHB, Lugner AK. Consequences of restricted STI testing for young heterosexuals in the Netherlands on test costs and QALY losses. Health Policy. 2018;122(2):198-203.

31. Tuite AR, Burchell AN, Fisman DN. Cost-effectiveness of enhanced syphilis screening among HIV-positive men who have sex with men: a microsimulation model. PLoS ONE. 2014;9(7):e101240.

32. Murray CJL. Global health statistics : a compendium of incidence, prevalence, and mortality estimates for over 200 conditions. Lopez AD, World Health O, World B, Harvard School of Public H, editors. Cambridge, MA : Cambridge, MA: Cambridge, MA : Published by Harvard University on behalf of the World Health Organization and the World Bank

Cambridge, MA : Distributed by Harvard University Press; 1996.

33. Mathers C, Fat DM, Boerma JT, World Health Organization. The global burden of disease 2004 update. Geneva, Switzerland: Geneva, Switzerland : World Health Organization; 2008.

34. Mathers C, Vos T, Stevenson C. The burden of disease and injury in Australia: Australian Institute of Health and Welfare; 1999.

35. Group PH. Victorian burden of disease study: Mortality and morbidity in 2001. Victorian Government Department of Human Services Melbourne, Australia; 2005.

36. Kwong JC, Crowcroft NS, Campitelli MA, Ratnasingham S, Daneman N, Deeks SL, Manuel FDG. Ontario Burden of Infectious Disease Study OAHPP/ICES Report. 2010.

37. Bijkerk P, van Lier A, McDonald S, Wallinga J, de Melker H. Appendix: State of infectious diseases in the Netherlands, 2013. Bilthoven: National Institute for Public Health and the Environment (RIVM); 2014 (Appendix RIVM report 150205001).

38. Kuznik A, Habib AG, Manabe YC, Lamorde M. Estimating the public health burden associated with adverse pregnancy outcomes resulting from syphilis infection across 43 countries in sub-Saharan Africa. Sexually Transmitted Diseases. 2015;42(7):369-75.

39. Liu Z, Wang T, Liu Y, Wang A, Xie D, Kong F, et al. Evaluating the progress to eliminate mother-to-child transmission (MTCT) of syphilis in Hunan Province, China: A study based on a health service delivery model. PLoS ONE. 2018;13(9):e0203565.

40. Russell WA, Owusu-Ofori S, Owusu-Ofori A, Micah E, Norman B, Custer B. Cost-effectiveness and budget impact of whole blood pathogen reduction in Ghana. Transfusion. 2021;61(12):3402-12.

41. Custer B, Agapova M, Martinez RH. The cost-effectiveness of pathogen reduction technology as assessed using a multiple risk reduction model. Transfusion. 2010;50(11):2461-73.

42. Husereau D, Drummond M, Petrou S, Carswell C, Moher D, Greenberg D, et al. Consolidated Health Economic Evaluation Reporting Standards (CHEERS) statement. BMC medicine. 2013;11:80.

43. Evers S, Goossens M, de Vet H, van Tulder M, Ament A. Criteria list for assessment of methodological quality of economic evaluations: Consensus on Health Economic Criteria. International Journal of Technology Assessment in Health Care. 2005;21(2):240-5.

44. Picot J, Copley V, Colquitt JL, Kalita N, Hartwell D, Bryant J. The INTRABEAM® Photon Radiotherapy System for the adjuvant treatment of early breast cancer: a systematic review and economic evaluation. Health technology assessment (Winchester, England). 2015;19(69):1-190.

45. The World Bank. World Bank Country and Lending Groups [Web Page]. Data Blog: The World Bank; 2021 [updated July 01, 2021. Available from: <https://blogs.worldbank.org/opendata/new-world-bank-country-classifications-income-level-2021-2022>.
